# Biometry challenges in the longest eyes we have encountered to date

**DOI:** 10.1016/j.ajoc.2024.101997

**Published:** 2024-01-14

**Authors:** Raul Plasencia-Salini, Amanda P. Havens, Kevin M. Miller

**Affiliations:** From the Stein Eye Institute and Department of Ophthalmology, David Geffen School of Medicine at UCLA, Los Angeles, CA, USA

**Keywords:** Intraocular lens power calculation, Cataract surgery, Ocular biometry, Axial length, High myopia

## Abstract

**Purpose:**

This report aims to present biometry challenges and solutions for a patient with the longest eyes we have encountered to date.

**Observations:**

A 41-year-old woman with a history of Crouzon syndrome, extreme axial myopia, and posterior segment staphylomas was referred for cataract evaluation. Optical biometry was attempted using two partial coherence interferometry and optical low-coherence reflectometry devices that were available in 2011. Neither device could measure the axial length (AL) of either eye, unfortunately. We were able to measure them by A scan ultrasound, however, with results of 40.59 mm for the right eye and 38.29 mm for the left eye. Shortly thereafter, she underwent uncomplicated phacoemulsification with posterior chamber intraocular lens implantation under topical anesthesia. Twelve years later, she returned for repeat optical biometry with 3 newer generation devices, 2 of which utilized swept-source optical coherence tomography (SS-OCT). Only 1 SS-OCT device, the Argos biometer, was able to obtain AL measurements, and they were 40.54 mm and 40.84 mm for the right and left eyes, respectively.

**Conclusions and importance:**

Biometry measurement using optical biometers on a patient with ALs greater than 40 mm was impossible in 2011 because of the relatively short gate for acceptable readings. Ultrasound biometry can also be challenging due to the presence of posterior staphylomas. However, a newer SS-OCT with a longer AL measurement capability enabled readings to be obtained more recently.

## 1 Introduction

Intraocular lens (IOL) power calculation can be challenging in very long eyes.^1^ Potential sources of error include staphylomas, for which the locus of best fixation may be uncertain; lens A constants and surgeon factors that may have been developed for eyes with normal ALs; and IOL power formulas that may be suboptimal for long eyes.^2^^,^^3^ It is generally known that optical biometry provides more precise measurement of AL in eyes with staphylomas because it measures from the corneal vertex to the locus of best fixation, which is often a shorter distance than the longest AL measureable.^3^ However, many commercially available optical biometers have a limited range in which they can measure, with electronic gates that stop at 38 mm.^4^

This report aims to present the problems of measuring ALs in a patient whose eyes were longer than this.

## 2 Case report

A 41-year-old woman with history of Crouzon syndrome, posterior segment staphylomas, myopic proptosis, prior tarsorrhaphies, and restrictive strabismus in both eyes was referred to one of the authors (KMM) for cataract evaluation in 2011. Her right eye had a corrected distance visual acuity (CDVA) of count fingers at 1 foot with a manifest refraction of −31.0 D and her left eye had a CDVA of 20/40^−2^ with a manifest refraction of −23.0 D.

During the preoperative visit, optical biometry measurements were attempted using 2 different instruments. The first was an IOLMaster 500 (Carl Zeiss Meditec AG, Jena, Germany), which is a partial coherence interferometry (PCI) device. The second was a Lenstar LS900 (Haag-Streit AG, Koeniz, Switzerland), which is an optical low-coherence reflectometry (OLCR) device. Interestingly to us at the time, neither device could measure the AL of either eye.

**Fig. 1:**
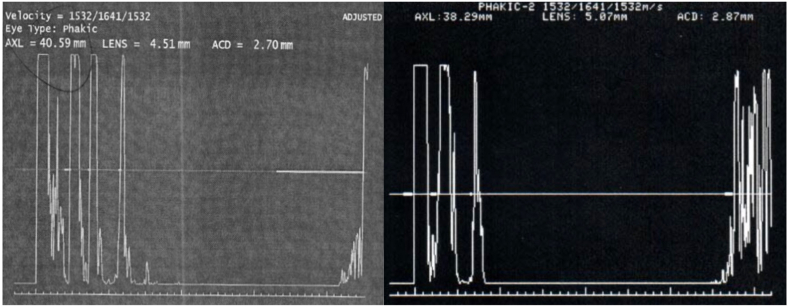
Preoperative immersion A scan ultrasound taken by the Eye Cubed V3 (Ellex, Minneapolis, Minnesota). Axial lengths measurements were 40.59 mm for the right eye by immersion and 38.29 mm for the left eye by applanation.

We were fortunately able to obtain AL measurements using the Eye Cubed V3 ultrasound device (Ellex, Minneapolis, Minnesota) in A-scan mode. The right eye measured 40.59 mm using an immersion technique and the left eye measured 38.29 mm using an applanation technique (Fig. 1). The reason for applanating the left eye was not recorded by the technician who performed the examination.

Keratometry readings were relatively flat at 38.2 D x 42.9 D for the right eye and 39.1 D x 42.1 D for the left eye. The patient was targeted for mild myopia using an adjusted SRK/T formula to avoid a hyperopic refractive outcome.^5^ For the right eye, a −7.0 D model AR40 M (Johnson & Johnson Vision, Santa Ana, California) 3-piece posterior chamber IOL was chosen with a target postoperative refraction of −2.08 D, while a −4.0 D model AR40 M IOL was chosen for the left eye with a target postoperative refraction of −1.77 D.

The patient underwent uncomplicated phacoemulsification with in-the-bag posterior chamber IOL implantation in both eyes under topical anesthesia. Surgeries were performed 1 month apart. To address preexisting corneal astigmatism, a single peripheral relaxing incision was placed in the steep corneal meridian of the right eye.

Following surgery, the patient measured a CDVA of 20/80^+2^ in her right eye with a manifest refraction of −2.50 + 3.00 × 180, and 20/25^−2^ in her left eye with a manifest refraction of −2.50 + 0.50 × 174. Over the subsequent years, she had regular follow-up visits with a retina specialist to monitor progression of myopic macular degeneration. She also began instilling glaucoma eye drops. Despite appropriate glaucoma therapy, the visual field defects of both eyes enlarged over the years.

The last refraction recorded in her medical chart, which was obtained on August 10, 2021, measured −3.00 + 4.75 × 157 in the right eye and −5.25 + 2.25 × 120 in the left eye, resulting in CDVAs of 20/125 and 20/50, respectively. During her last appointment, which was to a glaucoma specialist on September 15, 2023, her CDVAs were 2080 and 20/40 in the right and left eyes, respectively. Intraocular pressures were 13 mmHg in both eyes. Her visual fields were markedly constricted to less than 20° in both eyes.

**Fig. 2:**
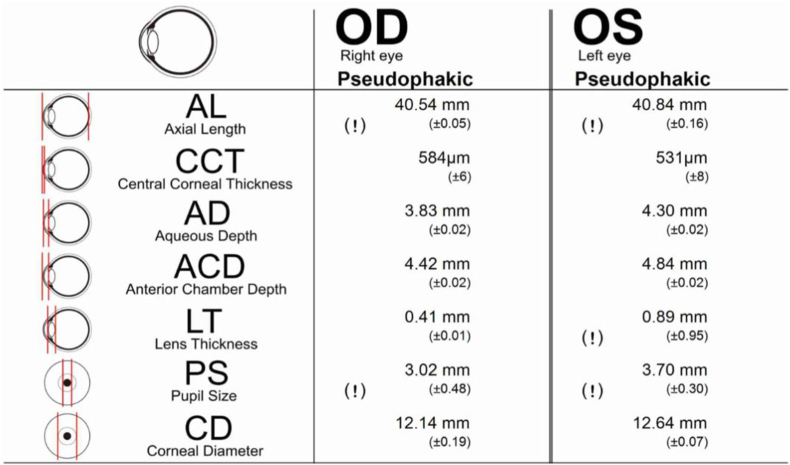
Postoperative Argos (Alcon Laboratories, Inc., Fort Worth, Texas, USA) SS-OCT measurements. Axial length measurements were 40.54 mm and 40.84 mm for the right and left eyes, respectively.

Twelve years after cataract surgery, in July 2023, she was invited to return for measurements with 3 newer generation optical biometry devices including the IOLMaster 700 (Carl Zeiss Meditec AG, Jena, Germany), which is a swept-source OCT (SS-OCT) device; the Argos (Alcon Laboratories, Inc., Fort Worth, Texas, USA), which is a SS-OCT device; and the Pentacam AXL Wave (Oculus Optikgeräte GmbH; Wetzlar, Germany), which is a Scheimpflug tomographer with built-in PCI capability for AL measurement. Of these 3 devices, only the Argos biometer was able to obtain readings on her eyes. It measured ALs of 40.54 mm and 40.84 mm for her right and left eyes, respectively (Fig. 2). The left eye reading was considerably longer than what had been measured 12 years earlier by A scan ultrasound. Interestingly, the Argos biometer is only rated for AL measurements between 14 and 38 mm.

## 3 Discussion

This report highlights the difficulty of obtaining AL measurements in eyes over 38 mm in length. We conducted a literature review on September 7, 2023 utilizing PubMed, Google Scholar, and ResearchGate using the key words biometry, axial length, longest eye, and degenerative myopia, but did not find any reports of human eyes longer than 40 mm. This patient is, therefore, quite unique.

It should be noted that the patient's left eye measured more than 2.5 mm longer after surgery by swept source optical methods than it had measured before surgery using an applanation A scan ultrasound technique. Possible explanations for this discrepancy include indentation of the cornea during the examination, aiming the ultrasound probe toward the side of a staphyloma, or axial elongation of the eye during the intervening years. The first two possibilities are more likely than the third given the lack of axial elongation of the right eye during the same time frame.

Cataract surgeons face several challenges when calculating IOL power in patients with extremely long eyes.^1^ One is the presence of posterior segment staphylomas. Because the locus of best eccentric fixation is not easily defined using immersion A scan ultrasonography, the functional AL is usually shorter than the distance from the corneal vertex to the back of a staphyloma, to which AL is often inadvertently measured.^6^ This results in the implantation of an IOL that is lower in power than required to achieve emmetropia, and thus a hyperopic refractive outcome, which is almost always disappointing to patients. B scan ultrasonography can be useful in extremely long eyes if the ultrasonographer knows the location of the locus of best eccentric fixation beforehand. Alternatively, optical biometry provides a more accurate measurement in eyes with staphylomas because it measures from the corneal vertex to the functional fovea.^3^ As we learned, however, this is not always possible in eyes with non-opaque cataracts if the AL is too high. Additional challenges of IOL power calculation in extremely long eyes are lens A constants and surgeon factors that were developed for eyes with normal ALs and IOL power formulas that may be suboptimal for extremely long eyes.^2^^,^^3^

It has been reported that optical biometers based on SS-OCT, such as the Argos and IOLMaster 700, are better able to measure AL through dense cataracts than biometers based on PCI and OLCR.^7^^,^^8^ Additionally, SS-OCT biometers have demonstrated excellent repeatability in several studies.^9^, ^10^, ^11^ Sabatino et al. found a highly positive correlation and strong agreement between the Argos and IOLMaster 700.^12^ Interestingly, Tamaoki et al. reported that the Argos biometer had a significantly better acquisition success rate than the IOLMaster 700.^13^ It should be noted that the manufacturers of optical biometers have not validated axial length measurements greater than 38 mm, which is where the gate is usually placed.^14^

The newer biometers don't all work the same. The IOLMaster 700 uses an equivalent refractive index for the entire eye, as do older biometers, while the Argos uses different refractive indices for each segment of the eye. This may be more accurate for eyes with long ALs, where the occupancy ratio of the crystalline lens is significantly lower.^13^^,^^15^ This methodology, known as the sum-of-segments, measures each segment of the eye at the correct velocity, similar to A-scan biometry, using specific indices of refraction, including 1.375 for the cornea, 1.336 for the aqueous and vitreous humors, and 1.41 for the lens in the phakic state.^15^ In our opinion, further studies are required to determine the repeatability and reproducibility of this new technology in eyes with extreme ALs.

Over the past decade, significant progress has been made developing methods to adjust AL measurements, which have improved the prediction accuracy of IOL formulas.^16^, ^17^, ^18^ Moreover, numerous studies have demonstrated that fourth generation formulas perform well in the subset of patients with high axial myopia.^2^^,^^19^, ^20^, ^21^ Unfortunately, these advancements were published after the patient's surgery had already performed. In this patient, the surgeon targeted the patient for mild myopia using the SRK/T formula based on his analysis of negative power lens implantation from his own published case series.^5^^,^^22^

Finally, high myopia can result in severe complications such as retinal detachment, so regular follow-up examination is recommended after cataract surgery for such patients.^23^, ^24^, ^25^ In addition, Crouzon syndrome may lead to optic atrophy, resulting in visual fields that are degraded in a concentric manner, which was observed in our patient.^26^

Despite an extensive literature search, no reported cases of cataract surgery in eyes with ALs greater than 40 mm were found. The longest AL reported we could find was by Vassallo et al. who measured 38.34 mm for the right eye and 38.31 mm for the left eye using A-scan ultrasonography in a patient with degenerative myopia.^27^ One of the authors (KMM) polled leading experts in IOL power calculation including Jack T. Holladay, MD, Douglas D. Koch, MD, Kenneth J. Hoffer, MD, H. John Shammas, MD, and Warren E. Hill, MD, about their experiences with long eyes. None could recall an eye longer than 37.5 mm (KMM, personal communications). An additional observation in this case is that the Argos biometer revealed significant differences in corneal diameter and anterior chamber depth between the two eyes. We can only speculate that these differences are related to the facial asymmetry that can be observed in Crouzon syndrome.

## 4 Conclusions

Accurate measurement of axial length using PCI, OLCR, and even most SS-OCT devices on a patient with ALs greater than 40 mm was challenging because an electronic gate at 38 mm blocks the measurement of longer eyes. Ultrasound measurement of AL can also be challenging due to the presence of posterior staphylomas. Only the Argos biometer could measure this patient, despite its reported 14–38 mm measurement capability.

## Patient Consent

Consent to publish the case report was not obtained. This report does not contain any personal information that could lead to the identification of the patient.

## Funding

No funding or grant support

## Authorship

All authors attest that they meet the current ICMJE criteria for Authorship.

## CRediT authorship contribution statement

**Raul Plasencia-Salini:** Data curation, Investigation, Writing – original draft, Writing – review & editing. **Amanda P. Havens:** Writing – review & editing. **Kevin M. Miller:** Conceptualization, Project administration, Supervision, Writing – original draft, Writing – review & editing.

## Declaration of competing interest

The authors declare the following financial interests/personal relationships which may be considered as potential competing interests: Kevin M. Miller, MD is a consultant for Alcon Laboratories. The other authors have no financial interests.

## References

[1] Cheng H., Liu L., Sun A., Wu M. (Jul 2020). Accuracy of modified axial length adjustment for intraocular lens power calculation in Chinese axial myopic eyes. Curr Eye Res.

[2] Zhang Y., Liang X.Y., Liu S., Lee J.W., Bhaskar S., Lam D.S. (2016). Accuracy of intraocular lens power calculation formulas for highly myopic eyes. J Ophthalmol.

[3] Shen P., Zheng Y., Ding X. (Feb 2013). Biometric measurements in highly myopic eyes. J Cataract Refract Surg.

[4] Sahin A., Hamrah P. (Jan 2012). Clinically relevant biometry. Curr Opin Ophthalmol.

[5] Kapamajian M.A., Miller K.M. (Feb 15 2008). Efficacy and safety of cataract extraction with negative power intraocular lens implantation. Open Ophthalmol J.

[6] Zaldivar R., Shultz M.C., Davidorf J.M., Holladay J.T. (May 2000). Intraocular lens power calculations in patients with extreme myopia. J Cataract Refract Surg.

[7] Shammas H.J., Ortiz S., Shammas M.C., Kim S.H., Chong C. (Jan 2016). Biometry measurements using a new large-coherence-length swept-source optical coherence tomographer. J Cataract Refract Surg.

[8] Huang J., Chen H., Li Y. (Feb 1 2019). Comprehensive comparison of axial length measurement with three swept-source OCT-based biometers and partial coherence interferometry. J Refract Surg.

[9] Nemeth G., Modis L. (Jun 2019). Ocular measurements of a swept-source biometer: repeatability data and comparison with an optical low-coherence interferometry biometer. J Cataract Refract Surg.

[10] Montes-Mico R. (Jan 1 2022). Evaluation of 6 biometers based on different optical technologies. J Cataract Refract Surg.

[11] Michael R., Wirkner K., Engel C., Loeffler M., Kirsten T., Rauscher F.G. (Jul 2023). Feasibility and repeatability of ocular biometry measured with IOLMaster 700 in a large population-based study. Ophthalmic Physiol Opt.

[12] Sabatino F., Matarazzo F., Findl O., Maurino V. (Aug 2019). Comparative analysis of 2 swept-source optical coherence tomography biometers. J Cataract Refract Surg.

[13] Tamaoki A., Kojima T., Hasegawa A. (2019). Clinical evaluation of a new swept-source optical coherence biometer that uses individual refractive indices to measure axial length in cataract patients. Ophthalmic Res.

[14] Zeiss. Iol Master 700 Technical Specifications..

[15] Shammas H.J., Taroni L., Pellegrini M., Shammas M.C., Jivrajka R.V. (Oct 1 2022). Accuracy of newer intraocular lens power formulas in short and long eyes using sum-of-segments biometry. J Cataract Refract Surg.

[16] Petermeier K., Gekeler F., Messias A., Spitzer M.S., Haigis W., Szurman P. (Sep 2009). Intraocular lens power calculation and optimized constants for highly myopic eyes. J Cataract Refract Surg.

[17] Wang L., Shirayama M., Ma X.J., Kohnen T., Koch D.D. (Nov 2011). Optimizing intraocular lens power calculations in eyes with axial lengths above 25.0 mm. J Cataract Refract Surg.

[18] Wang L., Koch D.D. (Nov 2018). Modified axial length adjustment formulas in long eyes. J Cataract Refract Surg.

[19] Bernardes J., Raimundo M., Lobo C., Murta J.N. (Mar 2021). A comparison of intraocular lens power calculation formulas in high myopia. J Refract Surg.

[20] Chen Y., Wei L., He W., Lu Y., Zhu X. (Oct 2021). Comparison of Kane, Hill-RBF 2.0, Barrett Universal II, and emmetropia verifying optical formulas in eyes with extreme myopia. J Refract Surg.

[21] Lin L., Xu M., Mo E. (Nov 2021). Accuracy of newer generation IOL power calculation formulas in eyes with high axial myopia. J Refract Surg.

[22] Abulafia A., Barrett G.D., Rotenberg M. (Mar 2015). Intraocular lens power calculation for eyes with an axial length greater than 26.0 mm: comparison of formulas and methods. J Cataract Refract Surg.

[23] Yao Y., Lu Q., Wei L., Cheng K., Lu Y., Zhu X. (Nov 1 2021). Efficacy and complications of cataract surgery in high myopia. J Cataract Refract Surg.

[24] Ikuno Y. (Dec 2017). Overview of the complications of high myopia. Retina.

[25] Ha A., Kim C.Y., Shim S.R., Chang I.B., Kim Y.K. (Apr 2022). Degree of myopia and glaucoma risk: a dose-response meta-analysis. Am J Ophthalmol.

[26] Gray T.L., Casey T., Selva D., Anderson P.J., David D.J. (Jun 2005). Ophthalmic sequelae of Crouzon syndrome. Ophthalmology.

[27] Vassallo JF T. (2020). The longest ocular axial length ever recorded?. Malta Med J.

